# *Bordetella hinzii* Meningitis in Patient with History of Kidney Transplant, Virginia, USA

**DOI:** 10.3201/eid2709.210350

**Published:** 2021-09

**Authors:** Joseph Pechacek, Jillian Raybould, Megan Morales

**Affiliations:** Virginia Commonwealth University Medical Center, Richmond, Virginia, USA

**Keywords:** meningitis/encephalitis, transplantation, bacteremia, *Bordetella hinzii*, bacteria, Virginia, United States

## Abstract

A patient in Virginia, USA, who had previously undergone multiple kidney transplantations showed signs of *Bordetella hinzii* bacteremia and meningitis. This emerging pathogen has been increasingly identified as a clinically significant pathogen in immunosuppressed and, less frequently, immunocompetent patients. This patient was treated and recovered without further issue.

Bordetella is a genus consisting mostly of strictly aerobic, small, gram-negative coccobacilli known to house the causative agent of pertussis and kennel cough in dogs (*Bordetella pertussis*) and cats (*B. bronchiseptica*). Over the past several decades, more human-derived clinical isolates have been identified, causing a range of pathologies. As our ability to rapidly and precisely identify clinical isolates grows, characterizing these rarer causes of human disease and their clinical significance and means of treatment has become vital.

*B. hinzii* was initially discovered in poultry isolates as a respiratory colonizer and cause of respiratory infection; subsequently, it was discovered to cause clinically significant infection in 1994 in a patient with advanced AIDS (*1*). Since this characterization, *B. hinzii* has been implicated as the cause of a range of clinical symptoms, including bacteremia (*1*–*3*), pulmonary disease (*4*–*6*), endocarditis (*7*), chronic cholangitis (*8*), and soft tissue abscess (*9*,*10*). We describe a case of meningitis in an immunocompromised patient that was caused by *B. hinzii*.

## Case Report

A 44-year-old man sought care in the emergency department of Virginia Commonwealth University Medical Center (Richmond, VA, USA) in June 2020 after experiencing 3 days of severe, diffuse headache, and subjective fevers; maximum measured temperature was 37.9°C. In 1986, the patient had undergone a living related donor kidney transplant for end-stage renal disease related to focal segmental glomerulosclerosis. He underwent another living related donor transplant in 1999 and a deceased-donor transplant in 2010 after the previous allografts failed. His most recent transplant, which occurred 10 years before the illness documented in this study, was performed with thymoglobulin induction and had been maintained with an immunosuppressive regimen of tacrolimus (goal trough of 5–7 ng/mL at the time of this hospitalization), 180 mg mycophenolic acid (2×/d), and 10 mg prednisone (1×/d). His medical history was further notable for seizure disorder and an allergy to penicillin, which manifested as severe urticaria.

He appeared stable in the emergency department; vital signs were temperature 37.5°C, pulse rate 90 bpm, respiratory rate 17 breaths/min, blood pressure 141/85 mm Hg, and oxyhemoglobin saturation 99% on room air. He reported neck stiffness associated with his headaches. When asked about animal exposures, he noted that he works in a warehouse and was regularly exposed to wild birds and rodents. Physical examination revealed discomfort but no signs of toxicity or neurologic deficits; cranial nerves were intact, and speech, motor abilities, and sensation were unremarkable. Complete blood count revealed a leukocyte count of 8.4 × 10^9^/L, hemoglobin of 11 g/dL, and platelet count of 163 × 10^9^/L. Additional bloodwork showed a glomerular filtration rate of 30 mL/min, which was consistent with the patient’s baseline given his history of chronic kidney disease. Noncontrasted computed tomography of the head demonstrated no acute pathology. Cerebrospinal fluid (CSF) was clear and colorless, and analysis indicated neutrophilic pleocytosis (Table 1). The patient was given 2 g intravenous (IV) ceftriaxone, a loading dose of 2 g IV vancomycin, and IV acyclovir. Ceftriaxone was rapidly replaced with 2 g meropenem given the patient’s immunosuppressed status and to empirically treat for the possibility of *Listeria*.

**Table 1:** Cerebrospinal fluid sample laboratory results in case of *Bordetella hinzii* meningitis in transplant patient, Virginia, USA*

Characteristic	Hospital admission	Hospital day 9
Leukocytes	852/mm^3^	26/mm^3^
PMNs	86%	3%
Monocytes	27%	1%
Lymphocytes	5%	96%
Erythrocytes	<1 per mm^3^	4 per mm^3^
Protein	149 mg/dL†	72 mg/dL†
Glucose	58 mg/dL‡	58 mg/dL§
Opening pressure	ND	39 cm H_2_O

*ND, not done; PMNs, polymorphonuclear leukocytes. †No serum protein available for comparison. ‡Serum glucose 108mg/dL. §Serum glucose 89mg/dL.

Additional CSF analysis was negative for cytomegalovirus PCR, enterovirus PCR, adenovirus PCR, herpes simplex virus 1 and 2 PCR, and cryptococcal antigen. Because of concerns about possible viral meningitis, we tested nasopharyngeal swab specimens by using the BioFire FilmArray 2.0 respiratory pathogen PCR panel (bioMérieux, https://www.biomerieux.com); results were negative. Acyclovir was stopped after CSF specimens tested negative for herpes simplex virus by PCR, and the patient continued on vancomycin and meropenem. On the second day of hospitalization, blood cultures taken at admission grew gram-negative rods; CSF culture grew gram-negative rods on the fourth day. Blood cultures drawn after antibiotics were administered showed no growth. Vancomycin was stopped on day 3 after cultures showed growth of only gram-negative organisms, which by that time had been identified by matrix-assisted laser desorption/ionization time-of-flight mass spectrometry as *Bordetella* spp. and subsequently *B. hinzii*. Oral ciprofloxacin (500 mg every 12 h) was added on day 6 of hospitalization because *B. hinzii* isolates have been reported to be sensitive to fluoroquinolones (*9*).

Around day 6 of hospitalization, the patient again began to experience intermittent headaches, although of lesser intensity than his initial symptoms. He underwent a second lumbar puncture on day 9, which revealed improving neutrophilic pleocytosis (Table 1). CSF cultures from this lumbar puncture remained negative.

The patient was discharged after demonstrating substantial improvement. However, because results of antibiotic sensitivity tests were still pending at that time, he was discharged on renally dosed IV meropenem (2 g every 12 h) and oral ciprofloxacin (500 mg every 12 h) for a planned total antibiotic duration of 21 days. Results of sensitivity testing of the *B. hinzii* isolates from blood and CSF samples returned after the patient was discharged revealed susceptibility to meropenem but only intermediate susceptibility to ciprofloxacin (Table 2). At follow-up 7 days after completion of therapy, the patient felt well and appeared to have clinically recovered.

**Table 2:** Antibiotic susceptibility of *Bordetella hinzii* isolate from blood and cerebrospinal fluid in transplant patient, Virginia, USA*

Antibiotic	Etest MIC from blood	Etest MIC from CSF
Ceftazidime	4 μg/mL	2 μg/mL
Ciprofloxacin	2 μg/mL	2 μg/mL
Imipenem	2 μg/mL	1 μg/mL
Meropenem	0.125 μg/mL	0.125 μg/mL
Piperacillin	1 μg/mL	1 μg/mL
Tobramycin	8 μg/mL	4 μg/mL
Trimethoprim/sulfamethoxazole	0.125 μg/mL	0.064 μg/mL

*Etest, bioMérieux (https://www.biomerieux.com). CSF, cerebrospinal fluid.

## Conclusions

Since its discovery as a cause of bacteremia in a patient with advanced AIDS in 1994 (*1*), *B. hinzii* has been implicated in a growing range of clinical syndromes as an opportunistic agent in immunocompromised and immunocompetent persons. It remains an infrequently isolated pathogen; further research and characterization is required. Its relatively recent recognition as an infective agent in humans is likely in part due to the increasingly common use of advanced identification techniques; in the past, *B. hinzii* might have been identified only at the genus level (*11*) or misidentified as a different, related bacterium (*1*)

*B. hinzii* is known to be found in the respiratory tracts of poultry as a colonizer and cause of respiratory infection (*12*). Exposure to poultry is an established risk factor for *B. hinzii* infection, especially in immunosuppressed populations (*6*). It is unclear whether this case-patient’s exposure to wild birds in his workplace constitutes a similar risk. Although *B. avium* is known to infect a range of wild and domesticated birds (*13*), whether *B. hinzii* affects birds other than poultry is not known. After the identification of *B. hinzii* from the respiratory tract of laboratory mice (*14*), rodents have also been proposed as potential reservoirs for this pathogen, and it has been isolated from wild mice (*11*) and rabbits (*12*). Definitive evidence of spread from an infected or colonized animal to a human has yet to be discovered.

The clinical isolates in this study were notable for sensitivity to carbapenems, piperacillin, and trimethoprim/sulfamethoxazole, which is in accordance with susceptibilities noted in other case reports on this species (*9*). We further noted an intermediate sensitivity to ciprofloxacin. Previous reports have indicated that levofloxacin might have a more favorable MIC for *B. hinzii* than ciprofloxacin (*9*).

Because *B. hinzii* is an emerging pathogen, its virulence factors require further research to be fully identified. Although the more classic *B. pertussis* (as well as *B. parapertussis* and *B. bronchiseptica*) rely on filamentous hemagglutinin and adenylate cyclase toxin as virulence factors, these proteins are absent in *B. hinzii* (*12*). These proteins are thought to assist in tracheal and pulmonary colonization in the more classic *Bordetella* spp. (*15*), so their absence from *B. hinzii* might begin to explain its propensity to cause syndromes atypical for bacteria of this genus.

In summary, we describe an unusual occurrence of *B. hinzii*–caused meningitis in an immunosuppressed patient. The clinical isolates in this study were sensitive to carbapenems, piperacillin, and trimethoprim/sulfamethoxazole but showed only intermediate sensitivity to ciprofloxacin. Clinicians should be aware of the possibility of human infection with this emerging pathogen, particularly in immunocompromised patients. 

## References

[1] Cookson BT, Vandamme P, Carlson LC, Larson AM, Sheffield JV, Kersters K, et al. Bacteremia caused by a novel Bordetella species, “*B. hinzii”.* J Clin Microbiol. 1994;32:2569–71. 10.1128/jcm.32.10.2569-2571.1994 7814500 PMC264104

[2] Kattar MM, Chavez JF, Limaye AP, Rassoulian-Barrett SL, Yarfitz SL, Carlson LC, et al. Application of 16S rRNA gene sequencing to identify *Bordetella hinzii* as the causative agent of fatal septicemia. J Clin Microbiol. 2000;38:789–94. 10.1128/JCM.38.2.789-794.2000 10655386 PMC86205

[3] Hristov AC, Auwaerter PG, Romagnoli M, Carroll KC. *Bordetella hinzii* septicemia in association with Epstein-Barr virus viremia and an Epstein-Barr virus-associated diffuse large B-cell lymphoma. Diagn Microbiol Infect Dis. 2008;61:484–6. 10.1016/j.diagmicrobio.2008.03.013 18482816

[4] Funke G, Hess T, von Graevenitz A, Vandamme P. Characteristics of *Bordetella hinzii* strains isolated from a cystic fibrosis patient over a 3-year period. J Clin Microbiol. 1996;34:966–9. 10.1128/jcm.34.4.966-969.1996 8815118 PMC228927

[5] Palacián Ruiz MP, Vasquez Martinez MA, Lopez Calleja AI. Respiratory infection caused by *Bordetella hinzii.* Arch Bronconeumol. 2013;49:409–10. 23755859 10.1016/j.arbres.2013.02.001

[6] Fabre A, Dupin C, Bénézit F, Goret J, Piau C, Jouneau S, et al. Opportunistic pulmonary *Bordetella hinzii* infection after avian exposure. Emerg Infect Dis. 2015;21:2122–6. 10.3201/eid2112.150400 26584467 PMC4672423

[7] González MM, Romano MPC, de Guzmán García Monge MT, Martín BB, García AS. *Bordetella hinzii* endocarditis, a clinical case not previously described. Eur J Case Rep Intern Med. 2019;6:000994. 30931262 10.12890/2019_000994 PMC6432829

[8] Arvand M, Feldhues R, Mieth M, Kraus T, Vandamme P. Chronic cholangitis caused by *Bordetella hinzii* in a liver transplant recipient. J Clin Microbiol. 2004;42:2335–7. 10.1128/JCM.42.5.2335-2337.2004 15131227 PMC404647

[9] Negishi T, Matsumoto T, Shinagawa J, Kasuga E, Horiuchi K, Natori T, et al. A case of cervical subcutaneous abscess due to *Bordetella hinzii.* Diagn Microbiol Infect Dis. 2019;95:114865. 10.1016/j.diagmicrobio.2019.07.003 31405631

[10] Kampmeier S, Rennebaum F, Schmidt H, Riegel A, Herrmann M, Schaumburg F. Peripancreatic abscess supported by *Bordetella hinzii.* New Microbes New Infect. 2020;34:100650. 10.1016/j.nmni.2020.100650 32025312 PMC6997295

[11] Jiyipong T, Morand S, Jittapalapong S, Raoult D, Rolain J-M. *Bordetella hinzii* in rodents, Southeast Asia. Emerg Infect Dis. 2013;19:502–3. 10.3201/eid1903.120987 23750354 PMC3647655

[12] Register KB, Kunkle RA. Strain-specific virulence of *Bordetella hinzii* in poultry. Avian Dis. 2009;53:50–4. 10.1637/8388-070108-Reg.1 19432003

[13] Harrington AT, Castellanos JA, Ziedalski TM, Clarridge JE III, Cookson BT. Isolation of *Bordetella avium* and novel Bordetella strain from patients with respiratory disease. Emerg Infect Dis. 2009;15:72–4. 10.3201/eid1501.071677 19116056 PMC2660683

[14] Hayashimoto N, Yasuda M, Goto K, Takakura A, Itoh T. Study of a *Bordetella hinzii* isolate from a laboratory mouse. Comp Med. 2008;58:440–6. 19004369 PMC2707133

[15] Mattoo S, Cherry JD. Molecular pathogenesis, epidemiology, and clinical manifestations of respiratory infections due to *Bordetella pertussis* and other Bordetella subspecies. Clin Microbiol Rev. 2005;18:326–82. 10.1128/CMR.18.2.326-382.2005 15831828 PMC1082800

